# Risk Factors Associated with Retinopathy of Prematurity in Very and Extremely Preterm Infants

**DOI:** 10.3390/medicina57050420

**Published:** 2021-04-27

**Authors:** Claudia Ioana Borțea, Florina Stoica, Marioara Boia, Emil Radu Iacob, Mihai Dinu, Roxana Iacob, Daniela Iacob

**Affiliations:** 1 Department of Neonatology, “Victor Babes” University of Medicine and Pharmacy, Eftimie Murgu Square 2, 300041 Timisoara, Romania; ioanabortea@yahoo.com (C.I.B.); marianaboia@yahoo.com (M.B.); danielariacob@yahoo.com (D.I.); 2 Department of Ophthalmology, Emergency Municipal Clinical Hospital, Gheorghe Dima Street 5, 300254 Timisoara, Romania; florinastoica@gmail.com; 3 Department of Pediatric Surgery, “Victor Babes” University of Medicine and Pharmacy, Eftimie Murgu Square 2, 300041 Timisoara, Romania; 4 Faculty of Medical Engineering, University “Politehnica” of Bucharest, Gheorghe Polizu Street 1-7, 011061 Bucharest, Romania; mihaidinu88@gmail.com; 5 Department of Radiology, “Victor Babes” University of Medicine and Pharmacy, Eftimie Murgu Square 2, 300041 Timisoara, Romania

**Keywords:** retinopathy of prematurity, risk factors, preterm birth, birth weight, artificial ventilation, very preterm infants, extremely preterm infants

## Abstract

*Background and Objectives*: Retinopathy of prematurity (ROP) is the leading cause of blindness in preterm infants. We studied the relationship between different perinatal characteristics, i.e., sex; gestational age (GA); birth weight (BW); C-reactive protein (CRP) and lactate dehydrogenase (LDH) concentrations; ventilation, continuous positive airway pressure (CPAP), and surfactant administration; and the incidence of Stage 1–3 ROP. *Materials and Methods*: This study included 247 preterm infants with gestational age (GA) < 32 weeks that were successfully screened for ROP. Univariate and multivariate binary analyses were performed to find the most significant risk factors for ROP (Stage 1–3), while multivariate multinomial analysis was used to find the most significant risk factors for specific ROP stages, i.e., Stage 1, 2, and 3. *Results*: The incidence of ROP (Stage 1–3) was 66.40% (164 infants), while that of Stage 1, 2, and 3 ROP was 15.38% (38 infants), 27.53% (68 infants), and 23.48% (58 infants), respectively. Following univariate analysis, multiple perinatal characteristics, i.e., GA; BW; and ventilation, CPAP, and surfactant administration, were found to be statistically significant risk factors for ROP (*p <* 0.001). However, in a multivariate model using the same characteristics, only BW and ventilation were significant ROP predictors (*p <* 0.001 and *p <* 0.05, respectively). Multivariate multinomial analysis revealed that BW was only significantly correlated with Stage 2 and 3 ROP (*p <* 0.05 and *p <* 0.001, respectively), while ventilation was only significantly correlated with Stage 2 ROP (*p <* 0.05). *Conclusions*: The results indicate that GA; BW; and the use of ventilation, CPAP, and surfactant were all significant risk factors for ROP (Stage 1–3), but only BW and ventilation were significantly correlated with ROP and specific stages of the disease, namely Stage 2 and 3 ROP and Stage 2 ROP, respectively, in multivariate models.

## 1. Introduction

Retinopathy of prematurity (ROP) is one of the main causes of visual impairment during childhood and the leading cause of blindness in preterm infants [1]. ROP is a proliferative vascular disease that affects the developing retina of preterm infants [2]. Clinical symptoms can range from spontaneous regression to complete bilateral detachment of the retina and blindness [3]. There are five stages of the disease, which denote its severity. Stage 1 (visible demarcation line between the vascularized and non-vascularized retina) and 2 ROP (visible ridge between the vascularized and non-vascularized retina) are considered mild, while Stage 3 (vessels emerging from the ridge), 4 (partial detachment of the retina), and 5 (complete detachment of the retina) are considered severe or treatment-requiring [4]. ROP (all stages) has been associated with a reduction of retinal sensitivity, while treatment-requiring ROP has also been associated with a reduction of retinal responsiveness [5]. The progress of ROP (all stages) and treatment-requiring ROP incidence rates over the past 10–20 years has varied across the world, with both having been shown to decrease in a very recent South Korean nationwide study [6], a non-significant change in the first but an increase in the latter between 2002 and 2011 has been shown in a study performed in Taiwan [7], and a rise in both has been reported in a study conducted in England between 1990 and 2011 [8].

ROP incidence is approximately 60% in preterm infants with a birth weight (BW) < 1500 g, consistent with most studies over time, while the rate of progression to severe ROP (Stages 3–5) has been reported to be approximately 15% [2,9]. The vast majority of severe ROP cases have been found in infants with a BW < 1251 g [10]. In addition to ROP, extremely low BW (<1000 g) has also been strongly associated with RDS (76% incidence) and a mortality rate of 55% in preterm infants [11]. Apart from the risk factors involved in the physiopathology of ROP, its incidence may be influenced by the gestational age (GA) of saved preterm infants [12]. According to the WHO, preterm infants can be classified based on the GA, namely, as moderate, very, and extremely preterm if the GA is 32–<37, 28–<32, and <28 weeks, respectively [13]. The tracheal intubation (TI) techniques used in neonatal intensive care units (NICUs) permit very and extremely preterm infant survival; however, without adequate control of the oxygen intake [4]. Recent studies have managed to reach a conclusion in regard to the optimal oxygenation that can permit the normal development of preterm infants without influencing the postnatal development of the retina [4]. In addition to oxygen administration [14,15], GA and BW represent major risk factors for ROP and are at the core of most ROP screening guides [16,17]. Studies on ROP physiopathology have suggested the possible implication of other factors, namely, maternal, such as preeclampsia and chorioamnionitis; prenatal; perinatal; demographic; genetic; and factors related to medical treatment and nutrition [16,18]. In a recent study, it has been demonstrated that prenatal steroid administration, GA, artificial ventilation, and respiratory distress syndrome (RDS) are correlated with ROP and bronchopulmonary dysplasia, intraventricular hemorrhage, and periventricular leukomalacia and the number of red cell concentrate transfusions are significantly correlated with ROP [2].

Understanding the mechanisms through which risk factors determine the onset and progress of ROP can improve the screening and treatment of this disease. One possible strategy is closely examining the connection between risk factors and different ROP stages. Although associations between ROP and various risk factors have been extensively researched over the past decades [19,20,21,22], there are fewer studies that are focused on the relationship between these risk factors and the different stages of the disease. In this study, we examined the correlation between multiple risk factors and ROP (Stage 1–3) incidence and analyzed which risk factors are more closely associated with specific stages of ROP. This report studies an East European population group and can contribute to major international studies on how both ROP and its various stages are linked to different risk factors and influenced by these.

## 2. Materials and Methods

### 2.1. Study Population

The study cohort included 247 very and extremely preterm infants that were admitted to the Neonatology Department of the Timişoara County Emergency Clinical Hospital between January 2017 and December 2019. The GA was calculated based on the last menstrual period and BW was measured during the first postnatal minutes. Levels of C-reactive protein (CRP), an inflammation marker, and lactate dehydrogenase (LDH), a hemolysis marker, were measured during the first six postnatal hours, usually prior to commencing artificial ventilation treatment and, in exceptional cases, during the first hour of ventilation treatment. CRP and LDH levels below 5 mg/L and 600 U/L, respectively, were considered normal, while levels above these thresholds were considered pathological. Surfactant was administered as a curative treatment for patients who were diagnosed with moderate or severe RDS from the first hours after birth. These patients required longer ventilation and CPAP treatment compared to those who were diagnosed with mild RDS, and this could have influenced the incidence and stage of ROP. ROP diagnostic examinations were performed by an experienced ophthalmologist using indirect ophthalmoscopy. Therapeutic interventions were immediately performed for all infants that developed Stage 3 ROP, and subsequently, none of the 247 patients included in this study developed Stage 4 and 5 ROP. Approval was obtained from the local Ethical Committee.

### 2.2. Statistical Analysis

Continuous variables are expressed as mean ± standard deviation (SD), while categorical variables are expressed as numbers and/or percentages. Univariate analysis was performed using the Student’s *t*-test and χ^2^ test to examine continuous and dichotomous variables, respectively. Univariate binomial logistic regression analysis was performed, and factors with a statistically significant correlation to ROP (Stage 1–3) were then analyzed by multivariate binomial logistic regression analysis, and the odds ratios (ORs) were calculated. These factors were then used in a multivariate multinominal logistic regression analysis to analyze the relationship between multiple independent factors and specific ROP stages, namely Stages 1, 2, and 3. All statistical tests have been 2-tailed and a *p*-value < 0.05 was considered statistically significant. All statistical analyses were performed using SPSS Version 26 (IBM Corporation, Armonk, NY, USA).

## 3. Results

### 3.1. Distribution of Patients

In this study, 247 infants with GA < 32 weeks and BW of ≤2560 g were screened for ROP. The male to female ratio was 1.09 (129 males and 118 females), with no statistically significant difference being found between sexes in regard to GA, BW, ventilation and CPAP duration, CRP and LDH concentrations, administration of surfactant, and ROP (Stage 1–3) incidence.

**Figure 1:**
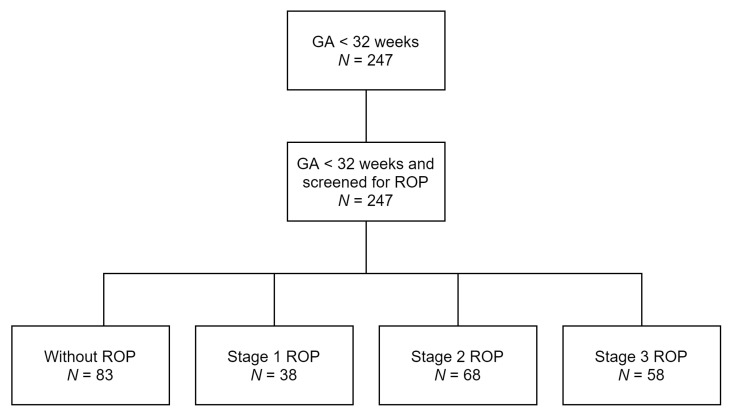
Distribution of infants in the study.

The incidence of ROP (Stage 1–3) in our study was 66.40% (164 infants) and 15.38% (38 infants), 27.53% (68 infants), and 23.48% (58 infants) for Stages 1, 2, and 3, respectively, as shown in the schematic diagram below (Figure 1).

Table 1 showcases the incidence of ROP (Stage 1–3) and specific stages in different subgroups of our study population. The patients were grouped based on various perinatal characteristics, such as sex; GA; BW; ventilation, CPAP, and surfactant treatment; and CRP and LDH levels. Most of the males (70.54%) and females (61.86%) were diagnosed with some stage (1–3) of ROP. The incidence of ROP (Stage 1–3) gradually decreased as GA and BA increased (100%, 91.67%, 84.21%, 76.92%, 65.79%, and 49.56% for 24–26, 27, 28, 29, 30, and 31 weeks, respectively, and 100.00%, 92.73%, 84.62%, and 50.00% for infants weighing <750, 750–999, 1000–1250, and >1250 g at birth, respectively. Infants that underwent ventilation, CPAP, and surfactant treatment showed much higher ROP (Stage 1–3) incidence rates (85.87%, 78.52%, and 83.18%, respectively) compared to those that did not undergo these treatments (54.84%, 51.79%, and 53.57%, respectively). Regardless of CRP and LDH levels being normal or pathological, the majority of infants showed higher ROP (Stage 1–3) incidence rates.

### 3.2. Univariate Comparison of Risk Factors

The perinatal characteristics of infants that developed and did not develop ROP (Stage 1–3) have been listed in Table 2. There were statistically significant differences between all the subgroups that developed the disease and the ones that did not, with the exception of infants that showed pathological CRP and LDH levels.

### 3.3. Univariate Analysis of Risk Factors and Their Correlation to ROP Incidence

A logistic regression analysis of each risk factor revealed that there were statistically significant differences between infants of different GAs, BWs, and treatment in regard to ROP (Stage 1–3) development (*p <* 0.001). As shown in Table 3, increased GA and BW were associated with decreased odds of exhibiting Stage 1–3 ROP (0.530, 95% CI from 0.417 to 0.673 and 0.997, 95% CI from 0.996 to 0.998, respectively), while undergoing artificial ventilation, CPAP, or surfactant treatment were associated with increased odds of exhibiting Stage 1–3 ROP (5.005, 95% CI from 2.570 to 9.746, 3.403, 95% CI from 1.957 to 5.918, and 3.403, 95% CI from 1.957 to 5.918, respectively). However, the differences between infants of different sexes, CRP levels, and LDH levels were not statistically significant (*p* = 0.150, *p* = 0.166, and *p* = 0.283, respectively). Therefore, following the independent analysis of these factors, ventilation treatment was the risk factor with the strongest correlation to ROP (Stage 1–3), followed by surfactant treatment, CPAP treatment, GA, and BW.

### 3.4. Multivariate Analyses

#### 3.4.1. Multivariate Analysis of Risk Factors and Their Correlation to ROP (Stage 1–3) Incidence

The perinatal characteristics mentioned in the previous section that were demonstrated to be significant ROP (Stage 1–3) risk factors, i.e., GA; BW; and ventilation, CPAP, and surfactant treatments were analyzed by multivariate binary logistic regression analysis, and the ORs were calculated. As seen in Table 4, in this model, only BW and ventilation remained statistically significant risk factors with *p <* 0.001 and *p <* 0.05, respectively, confirming that BW was negatively correlated with ROP (Stage 1–3), while ventilation was positively correlated with the disease. Out of the two factors, the BW had less impact on ROP (Stage 1–3) incidence and is, therefore, a less significant risk factor compared to ventilation treatment (0.998, 95% CI from 0.997 to 0.998 compared to 2.528, 95% CI from 1.082 to 5.904).

#### 3.4.2. Multivariate Analysis of Risk Factors and Their Correlation to Specific ROP Stages

The same perinatal characteristics mentioned in the previous section were analyzed by multivariate multinomial logistic regression analysis to study their relationship to specific stages of ROP, and the ORs were calculated. As presented in Table 5, in this model, none of the above-mentioned characteristics were significant risk factors for Stage 1 ROP, but BW was nearly significant (0.999, 95% CI from 0.997 to 1.000, *p* = 0.056). However, both BW and ventilation treatment were significant predictors for Stage 2 ROP (0.998, 95% CI from 0.997 to 0.999, *p* = 0.005 and 3.239, 95% CI from 1.241 to 8.455, *p* = 0.016), as infants with a high BW were less likely to have ROP and those who underwent ventilation treatment were more likely to have Stage 2 ROP. The only significant predictor of Stage 3 ROP was BW (0.997, 95% CI from 0.995 to 0.998, *p <* 0.001).

**Figure 2:**
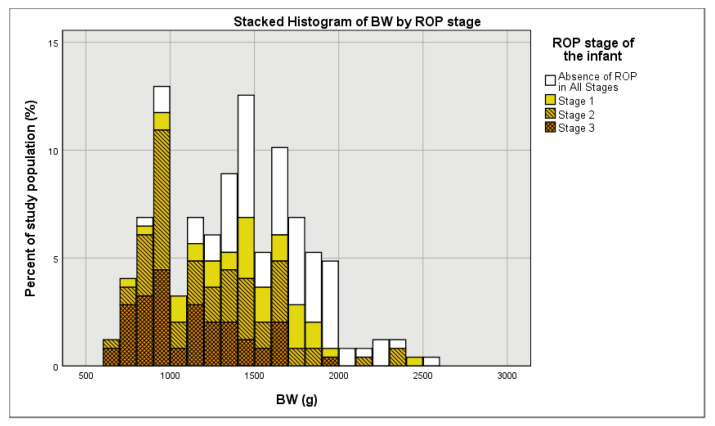
Distribution of BW (in 100 g increments) stratified by ROP stage.

**Figure 3:**
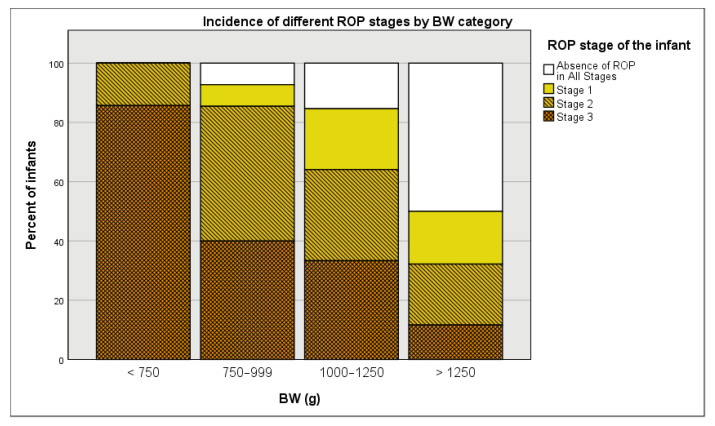
Percent incidence of various ROP stages stratified by BW groups (<750, 750–999, 1000–1250, and >1250 g).

As expected, in this study, BW has been found to be a nearly significant predictor of Stage 1 ROP and a significant predictor of Stage 2 and 3 ROP. In Figure 2, a stacked histogram of BW by ROP stage, it is noticeable that as BW increased, there was an increase in the percent of mild and inexistent ROP cases and a decrease in the percent of severe ROP cases. The same trend can be noticed when using four different BW groups, namely <750, 750–999, 1000–1250, and >1250 g (Figure 3).

**Figure 4:**
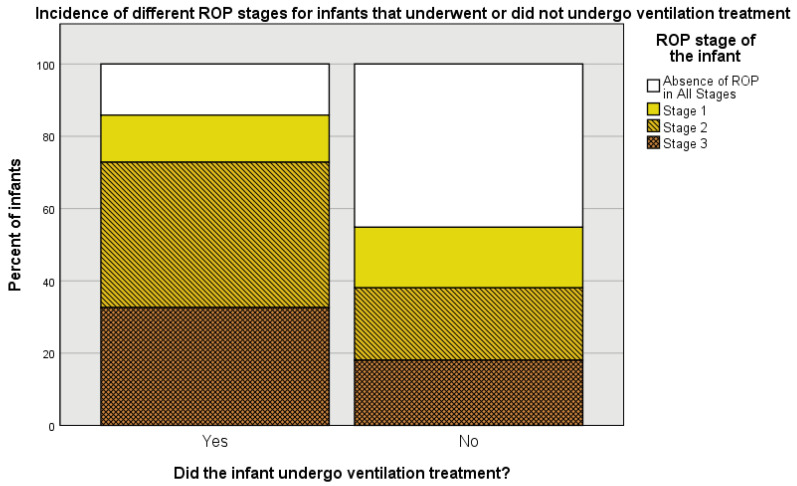
Percent incidence of various ROP stages for infants that underwent and did not undergo ventilation treatment.

Artificial ventilation treatment, another well-known ROP risk factor, has been found to be a significant predictor of Stage 2 ROP, but it did not have statistical significance as a predictor of Stage 1 and 3 ROP. In Figure 4, it is noticeable that Stage 2 ROP incidence is much higher and the percent of infants without ROP is much lower for infants that received ventilation treatment compared to those that did not.

## 4. Discussion

The primary purpose of this report was to analyze whether differences in perinatal characteristics alter the incidence of ROP (Stage 1–3) and specific ROP stages, namely Stages 1, 2, and 3 ROP. The latter has not been as extensively analyzed and discussed in the literature as the former, with most studies focusing on the incidence of ROP (all stages) rather than specific ROP stages. There has been extensive research on ROP incidence in the past years; however, there is yet no consensus regarding which risk factors are the more significant predictors of ROP or its various stages, nor on the underlying mechanisms through which these risk factors influence the onset and severity of ROP [16]. In addition to well-known risk factors, such as GA and BW [17,23]; oxygen and surfactant administration [24,25], including mechanical ventilation [22]; and blood transfusion [21], recent studies have shown that slow weight gain [26], average day length [27], bronchopulmonary dysplasia [2,28], and inhaled nitric oxide [29] are also strongly correlated with ROP. Moreover, certain perinatal characteristics of infants, such as intrauterine hypoxia, necrotizing enterocolitis, and hemolytic disease, have been reported to be inversely associated with ROP [22]. Our study confirmed BW is one of the most closely associated factors with ROP, as it was the only factor we studied that showed a significant association with both Stage 2 and 3 ROP, as well as a near-significant association with Stage 1 ROP, while ventilation was not significantly associated with Stage 1 and 3 ROP. Therefore, in similar models that exclude Stage 4 and 5 ROP due to therapeutic interventions stopping further development of the disease, BW may be a better predictor for ROP than ventilation, as it is associated with more specific stages of the disease. Other studies have also reported that BW is the most reliable predictor of ROP (any stage) when using a multivariate model [21], as well as a more statistically significant risk factor than ventilation [28,29]. However, contrary to most reports in the literature [2,12,24,26,27,28], our results did not reveal any significant relationship between GA and ROP (Stage 1–3) in a multivariate model. GA was inversely correlated with ROP in the univariate analysis but the statistical significance was diminished following the inclusion of other factors into the model, showing that BW and ventilation performed better in our multivariate model. This may be due to our study including only very and extremely preterm infants, while some studies have included infants with GA ≥ 32 weeks [24,28] or have not specified the GA upper limit and may include infants with GA ≥ 32 weeks [26,27] and, therefore, the difference between groups with a high GA and those with a low GA in regard to ROP incidence may be better contrasted and the relationship between GA and ROP may be better emphasized in those cases.

CPAP treatment has been shown to be significantly correlated with ROP (any stage) in a multivariate model [24]; however, in our study, it was only significantly correlated with ROP (Stage 1–3) in univariate models.

In this study, surfactant treatment, which has also been reported as a statistically significant ROP risk factor [25], was administered only curatively for patients diagnosed with moderate or severe RDS from the first hours after birth. Therefore, unlike most studies, in which the majority or all of the infants included undergo prophylactic surfactant treatment and a comparison with a control group cannot be performed, the largest part (56.68%) of the infants included in this study did not undergo surfactant treatment. However, although there was a significant difference between the group which underwent surfactant treatment and the group that did not undergo the treatment in regard to ROP (Stage 1–3) incidence in our univariate model, no significant differences were found in our multivariate model.

The larger the number of perinatal factors taken into account, the more ROP risk factors can be associated with ROP, and their respective levels of correlation with ROP, as well as the statistical significance of these correlations, can be better assessed. This is due to the interlinked mechanisms between risk factors, and any new risk factor that is identified improves the statistical model and can ultimately assist ophthalmologists in ROP diagnosis, prediction, and treatment [30]. Therefore, the complex interplay between different perinatal characteristics requires further research in order to ascertain how these factors impact ROP onset and progress and improve clinical practice guidelines for ROP.

To our knowledge, LDH and CRP levels have been scarcely studied as possible risk factors for ROP. Although our results did not show any strong relationship between these two markers and ROP, these may be revealed to be significant in future studies that include larger cohorts and Stage 4 and 5 ROP.

The main limitation of our study is the low number of perinatal characteristics recorded. As seen in our results, the statistical significance of a risk factor changes when an additional variable is taken into account, and, given the complex inter-relationships between these characteristics and ROP, the more potential risk factors are considered in the analysis, the more accurately this depicts the correlation of independent risk factors to ROP incidence. The absence of Stage 4 and 5 ROP in this study is another possible limitation, as the addition of other stages of the disease would influence the statistical model and, therefore, the p-values would be different for all the correlations mentioned above. A third limitation may be that all the patients included in this study had a GA < 32 weeks and, therefore, ROP can be more clearly associated with lower GA in models where infants with a higher GA were included.

## 5. Conclusions

This study confirms several known ROP risk factors, i.e., GA; BW; and ventilation, CPAP, and surfactant administration, which were all significant risk factors for ROP (Stage 1–3) in a univariate statistical model, but in multivariate models, only BW and ventilation are significant predictors of ROP (Stage 1–3) and specific stages of the disease, namely Stage 2 and 3 ROP and Stage 2 ROP, respectively.

## Figures and Tables

**Table 1:** Distributions of the various ROP groups by perinatal characteristics.

		ROP (Stage 1–3)		Stage 1 ROP		Stage 2 ROP		Stage 3 ROP	
Characteristics	Population (*n*)	Cases (*n*)	Percent of Total Population	Cases (*n*)	Percent of Total Population	Cases (*n*)	Percent of Total Population	Cases (*n*)	Percent of Total
**Total**	247	164	66.40%	38	15.38%	68	27.53%	58	23.48%
**Sex**									
Male	129	91	70.54%	21	16.28%	40	31.01%	30	23.26%
Female	118	73	61.86%	17	14.41%	28	23.73%	28	23.73%
**GA (weeks)**									
24	3	3	100.00%	0	0.00%	2	66.67%	1	33.33%
25	6	6	100.00%	0	0.00%	2	33.33%	4	66.67%
26	11	11	100.00%	0	0.00%	5	45.45%	6	54.55%
27	12	11	91.67%	1	8.33%	5	41.67%	5	41.67%
28	38	32	84.21%	9	23.68%	10	26.32%	13	34.21%
29	26	20	76.92%	1	3.85%	8	30.77%	11	42.31%
30	38	25	65.79%	5	13.16%	12	31.58%	8	21.05%
31	113	56	49.56%	22	19.47%	24	21.24%	10	8.85%
**BW (g)**									
<750	7	7	100.00%	0	0.00%	1	14.29%	6	85.71%
750–999	55	51	92.73%	4	7.27%	25	45.45%	22	40.00%
1000–1250	39	33	84.62%	8	20.51%	12	30.77%	13	33.33%
>1250	146	73	50.00%	26	17.81%	30	20.55%	17	11.64%
**Ventilation**									
Yes	92	79	85.87%	12	13.04%	37	40.22%	30	32.61%
No	155	85	54.84%	26	16.77%	31	20.00%	28	18.06%
**CPAP**									
Yes	135	106	78.52%	14	10.37%	47	34.81%	45	33.33%
No	112	58	51.79%	24	21.43%	21	18.75%	13	11.61%
**Surfactant**									
Yes	107	89	83.18%	15	14.02%	40	37.38%	34	31.78%
No	140	75	53.57%	23	16.43%	28	20.00%	24	17.14%
**CRP**									
Pathological	72	53	73.61%	12	16.67%	23	31.94%	18	25.00%
Normal	175	111	63.43%	26	14.86%	45	25.71%	40	22.86%
**LDH**									
Pathological	159	109	69.19%	26	15.70%	48	29.07%	35	24.42%
Normal	88	55	60.00%	12	14.67%	20	24.00%	23	21.33%

Abbreviations: GA, gestational age; BW, birth weight; CPAP, continuous positive airway pressure; CRP, C-reactive protein; LDH, lactate dehydrogenase; ROP, retinopathy of prematurity.

**Table 2:** Univariate comparison of perinatal characteristics in infants who developed ROP (Stage 1–3) and those who did not.

Characteristics	Without ROP (Any Stages) (*n* = 83)	With ROP Stage 1–3 (*n* = 164)	*p*-Value
Male	38 (45.78%)	91 (55.49%)	<0.001
Female	45 (54.22%)	73 (44.51%)	0.013
GA, weeks	30.4 ± 1	29.1 ± 1.9	<0.001
BW, g	1620 ± 332	1234 ± 373	<0.001
Ventilation treatment	13 (15.66%)	79 (48.17%)	<0.001
CPAP treatment	29 (34.94%)	106 (64.63%)	<0.001
Surfactant treatment	18 (21.69%)	89 (54.27%)	<0.001
CRP, mg/dL	4.57 ± 6.2	6.12 ± 8.92	0.158
LDH, U/L	878 ± 518	981 ± 782	0.279

Abbreviations: GA, gestational age; BW, birth weight; CPAP, continuous positive airway pressure; CRP, C-reactive protein; LDH, lactate dehydrogenase; ROP, retinopathy of prematurity. The above categorical and continuous data are displayed as *n* (%) or means ± SD, respectively.

**Table 3:** Univariate binary logistic regression analysis results. GA, BW, Ventilation, CPAP, and surfactant treatment were found to be significant ROP (Stage 1–3) risk factors.

Characteristics	OR (95% CI)	*p*-Value	ROP (Stage 1–3) Incidence Rate *
Sex, male/female	1.476 (0.869–2.509)	0.150	70.54%/61.86%
GA, weeks	0.530 (0.417–0.673)	<0.001	
BW, g	0.997 (0.996–0.998)	<0.001	
Ventilation, yes/no	5.005 (2.570–9.746)	<0.001	85.87%/54.84%
CPAP, yes/no	3.403 (1.957–5.918)	<0.001	78.52%/51.79%
Surfactant, yes/no	4.285 (2.338–7.853)	<0.001	83.18%/53.57%
CRP, mg/dL	1.028 (0.989–1.070)	0.166	
LDH, U/L	1.000 (1.000–1.001)	0.283	

Abbreviations: GA, gestational age; BW, birth weight; CPAP, continuous positive airway pressure; CRP, C-reactive protein; LDH, lactate dehydrogenase; OR, odds ratio; CI, confidence interval; ROP, retinopathy of prematurity. ORs are displayed as crude values and include only the first subcategory in the case of sex, ventilation, CPAP, and surfactant treatment, all dichotomous variables. * Incidence rate of ROP among patients when their sex is male/female and when they underwent/did not undergo ventilation, CPAP, and surfactant treatment.

**Table 4:** Multivariate binary logistic regression analysis results. In contrast with univariate analysis of the same factors, only BW and ventilation were found to be significant risk factors for ROP (Stage 1–3).

Characteristics	OR (95% CI)	*p*-Value
GA, weeks	0.863 (0.630–1.182)	0.358
BW, g	0.998 (0.997–0.999)	<0.001
Ventilation, yes/no	2.528 (1.082–5.904)	0.032
CPAP, yes/no	1.240 (0.616–2.496)	0.547
Surfactant treatment, yes/no	0.923 (0.393–2.168)	0.855

Abbreviations: GA, gestational age; BW, birth weight; CPAP, continuous positive airway pressure; OR, odds ratio. ORs are displayed as crude values and include only the first subcategory in the case of ventilation, CPAP, and surfactant treatment, all dichotomous variables.

**Table 5:** Comparisons between infants with an absence of ROP in all stages and those with Stage 1, 2, and 3 ROP. In this model, only BW was a significant predictor of Stage 3 ROP (*p <* 0.001), while both ventilation and BW were significant predictors of Stage 2 ROP (*p <* 0.05).

Characteristics	OR (95% CI)	*p*-Value
**Stage I ROP**		
GA, weeks	1.005 (0.668–1.513)	0.980
BW, g	0.999 (0.997–1.000)	0.056
Ventilation, yes/no	1.835 (0.601–5.599)	0.286
CPAP, yes/no	0.543 (0.207–1.424)	0.215
Surfactant treatment, yes/no	1.524 (0.481–4.828)	0.474
**Stage II ROP**		
GA, weeks	0.896 (0.630–1.276)	0.544
BW, g	0.998 (0.997–0.999)	0.005
Ventilation, yes/no	3.239 (1.241–8.455)	0.016
CPAP, yes/no	1.602 (0.695–3.691)	0.268
Surfactant treatment, yes/no	0.955 (0.348–2.619)	0.928
**Stage III ROP**		
GA, weeks	0.789 (0.546–1.138)	0.205
BW, g	0.997 (0.995–0.998)	<0.001
Ventilation, yes/no	2.156 (0.731–6.362)	0.164
CPAP, yes/no	2.216 (0.865–5.682)	0.098
Surfactant treatment, yes/no	0.503 (0.162–1.565)	0.236

Abbreviations: ROP, retinopathy of prematurity; GA, gestational age; BW, birth weight; CPAP, continuous positive airway pressure; OR, odds ratio; CI, confidence interval. ORs are displayed as crude values and include only the first subcategory in the case of ventilation, CPAP, and surfactant treatment, all dichotomous variables.

## Data Availability

All data showcased in this study can be obtained on request from the corresponding author. The data have not been publicized in order to meet the requirements of the European Union General Data Protection Regulation and limit publically-available personal information of the infants.
